# The Feasibility of Health Trainer Improved Patient Self-Management in Patients with Low Health Literacy and Poorly Controlled Diabetes: A Pilot Randomised Controlled Trial

**DOI:** 10.1155/2016/6903245

**Published:** 2016-10-19

**Authors:** Joanne Protheroe, Trishna Rathod, Bernadette Bartlam, Gillian Rowlands, Gerry Richardson, David Reeves

**Affiliations:** ^1^Research Institute for Primary Care & Health Sciences, Keele University, Keele, UK; ^2^Section for Health Promotion and Health Services, Institute for Public Health, Aarhus University, Aarhus, Denmark; ^3^Institute of Health and Society, University of Newcastle, Newcastle upon Tyne, UK; ^4^Centre for Health Economics, University of York, York, UK; ^5^Centre for Primary Care and Centre for Biostatistics, Manchester Academic Health Science Centre (MAHSC), University of Manchester, Manchester, UK

## Abstract

Type 2 diabetes mellitus is most prevalent in deprived communities and patients with low health literacy have worse glycaemic control and higher rates of diabetic complications. However, recruitment from this patient population into intervention trials is highly challenging. We conducted a study to explore the feasibility of recruitment and to assess the effect of a lay health trainer intervention, in patients with low health literacy and poorly controlled diabetes from a socioeconomically disadvantaged population, compared with usual care.* Methods*. A pilot RCT comparing the LHT intervention with usual care. Patients with HbA1c > 7.5 (58 mmol/mol) were recruited. Baseline and 7-month outcome data were entered directly onto a laptop to reduce patient burden.* Results*. 76 patients were recruited; 60.5% had low health literacy and 75% were from the most deprived areas of England. Participants in the LHT arm had significantly improved mental health (*p* = 0.049) and illness perception (*p* = 0.040). The intervention was associated with lower resource use, better patient self-care management, and better QALY profile at 7-month follow-up.* Conclusion*. This study describes successful recruitment strategies for hard-to-reach populations. Further research is warranted for this cost-effective, relatively low-cost intervention for a population currently suffering a disproportionate burden of diabetes, to demonstrate its sustained impact on treatment effects, health, and health inequalities.

## 1. Introduction

Diabetes mellitus (DM) is a disorder of glucose metabolism suffered by over 4 million UK people, 90 per cent of whom have Type 2 diabetes [1, 2]. Type 2 diabetes is more common in middle-aged or older people and greatest in deprived communities [3]. The risk of developing Type 2 diabetes can be reduced by lifestyle modification [4]. Deprivation is strongly associated with increased levels of obesity, physical inactivity, unhealthy diet, smoking, and poor blood pressure control, all potentially modifiable factors and all associated with an increased risk of developing diabetes or the risk of developing complications in people with diabetes [5]. The risk of developing complications such as heart disease, stroke, renal failure, and blindness is strongly linked to the tightness of glycaemic control [6, 7]. Patients' knowledge about diabetes and how to manage it, together with lifestyle choices, is central to the tightness of glycaemic control [8, 9].

Limited health literacy and numeracy skills are more common in areas of socioeconomic deprivation [10]. If this is linked with evidence that low health literacy is independently associated with worse glycaemic control and higher rates of diabetic complications, inadequate health literacy may be a significant factor in the disproportionate burden of diabetes and diabetes-related complications in more socioeconomically disadvantaged populations [11]. Studies have shown that people with low health literacy have lower levels of self-management of chronic disease, including poorer diabetes self-management [12–14].

Individuals with inadequate health literacy are less likely to be recruited into research studies or randomised controlled trials of healthcare interventions [15, 16]. This selection bias common to trials of interventions, may have implications for the likelihood of uptake of traditional diabetes educational interventions [17, 18]. In summary, we have a population of people with Type 2 diabetes and low health literacy at increased risk of complications who may be ill-served by the currently available diabetes educational self-management programmes.

Lay health trainers (LHTs) are a UK government initiative using peer or lay educators, living in the local community, designed to reduce health inequalities by engaging with and focusing on deprived or hard-to-reach populations [19, 20]. They are intended to promote affordable, practical, socioculturally relevant lifestyle advice within communities.

This study aims to

1. develop a LHT intervention to encourage patients to make healthy lifestyle choices in the management of Type 2 DM. This intervention is intended to improve patient self-management of their diabetes;
2. to explore the feasibility of recruitment of patients, with low health literacy and poorly controlled diabetes from a socioeconomic disadvantaged population, to a trial of a LHT intervention;
3. to collect data on a range of outcome measures and look for provisional indications of effectiveness and cost-effectiveness of the LHT intervention, in order to inform the design of a subsequent large-scale randomised controlled trial.

## 2. Participants and Methods

### 2.1. Sample

We conducted a pilot randomised controlled trial comparing the LHT intervention with usual care. Patients were recruited from six family doctor practices in Blackpool, from October 2012 to September 2013. Blackpool is the 6th most deprived local authority area in England and has a high prevalence of diabetes Type 2 (8.3%) compared with a national average of 5.8% [21, 22]. Patients who were aged over 18 years with poorly controlled diabetes (HbA1c > 7.5 or 58 mmol/mol in at least the last 2 measures) were eligible to be recruited. Those who were deemed ineligible by the practice staff (usually because of being too ill or too cognitively impaired to participate) were excluded.

Patients were identified and contacted by the practice nurse and informed about the study. Interested patients then had their contact details forwarded to the study research nurse. The research nurse contacted the patient to arrange an appointment to discuss the trial in depth at a face-to face meeting, either at home or at the practice as preferred by the patient. Consenting participants completed the baseline questionnaire and were randomised to one of the two trial arms (usual care or LHT).

Initially, potential participants were identified by the practice nurse at their routine review appointment from four family doctor practices. This yielded a poorer than expected recruitment and so the recruitment method was changed such that the practice nurse identified all potentially eligible patients and telephoned them to see if they would be interested in the study, rather than seeing the patient opportunistically at their routine check-up. Furthermore, an additional two family doctor practices were recruited to the trial.

### 2.2. Intervention

The intervention consisted of a structured interview with the LHT and development of an individualised patient self-management plan, plus up to three two-monthly support phone calls from the LHT (depending on agreements between the patient and the LHT) for a maximum of 6 months.

The structured interview supported the patient to identify areas where they could improve their health and used a locally developed menu (by collating existing locally available options) of support options available to that patient. Literacy skills teaching was not part of the intervention, but the LHT had information to enable them to, on request, signpost patients towards basic skills courses in their locality. The LHT did not provide medical or nursing advice. If the patient asked the LHT medical questions, patients were referred back to the practice nurse or family doctor. The LHTs had received training from the research team on evidence based diabetes care and appropriate lifestyle advice. In addition to providing information and advice aimed at changing key beliefs such as perceptions of risk from diabetes and the advantages and disadvantages of behaviour change, the LHTs advised them about essential health care tests and checks they should receive regularly as advised by Diabetes UK (blood pressure, cholesterol, feet and eye examinations, etc.). Using expertise from the research team, NHS Blackpool and Diabetes UK, the Wellness Service, employing the LHTs, developed a pamphlet designed for individuals with low health literacy to manage their diabetes [23].

Patients randomised to the control group received usual medical care. In the UK, usual care management of diabetes involves the family doctor practice keeping a register of all patients diagnosed with diabetes and usually inviting those patients into the practice for a review at least every 12 months. At this yearly review, usually led by the practice nurse, patients will be monitored and the following care processes should be undertaken: BMI measurement; BP measurement; haemoglobin A1c (HbA1c) measurement; cholesterol measurement; record of smoking status; foot examination; albumin: creatinine ratio; serum creatinine measurement.

### 2.3. Data Collection and Outcome Measures

To reduce the burden on the participant, the baseline demographics and outcomes were collected face-to-face by a research nurse who entered responses directly onto a security encrypted laptop. Outcomes were assessed at 7 months after randomisation via a telephone call from a different research nurse. Baseline demographics collected were age, gender, deprivation, health literacy, marital status, employment, ethnicity, and education. One aim of the pilot trial was to assess a broad spectrum of outcome measures (several of which overlap in health domains) for the purpose of selecting the most suitable subset for a larger trial in this population. Outcome measures included validated measures of diabetes self-care and quality of life, diabetes services and checks, EQ5D, mental well-being, illness perception, mental and physical health, resource use, and HbA1c values.

*Deprivation*. The index of multiple deprivation (IMD) 2010 is a measure of deprivation for small areas in England. It ranks areas from 1, the most deprived area, to 32,482 the least deprived area. The rank of deprivation is based on seven weighted domains: income; employment; education, skills, and training; health and disability; crime; barriers to housing and services; and living environment. Based on the participants' residential postcode, the rank of deprivation was obtained and then categorised into five groups with 1 being the most deprived area and 5 being the least deprived area in England [24].

*Health Literacy*. The Newest Vital Sign UK, validated for use with a UK population, was used to assess health literacy [25]. Participants were asked 6 questions based on a food label and a score of ≥4 was deemed to indicate adequate health literacy and a score <4 was deemed as less than adequate.

*Diabetes Self-Care*. It was measured using the Summary of Diabetes Self-Care Activities Measure [26]. This is a validated brief self-report questionnaire of diabetes self-management that assesses general diet, specific diet, exercise, blood-glucose testing, foot care, and smoking.

*Diabetes Quality of Life*. It was assessed using The Diabetes Quality of Life Brief Clinical Inventory [27]. This is a short 15-item scale that covers a broad range of issues ranging from patient satisfaction with their diabetes regimen to worries over diabetes symptoms and consequences.

*Diabetes UK Scale Items*. It is based on 9 questions (those applicable to primary care) out of 15 from Diabetes UK, relating to how many services and checks patients received to manage their diabetes; a total number of services and checks received was created [28].

*Health-Related Quality of Life*. It was assessed using the EQ5D which provides a measure of generic health-related quality of life [29]. This instrument enables the calculation of QALYs (quality adjusted life years), a composite measure of health obtained by weighing each period of follow-up time by the value corresponding to the health-related quality of life (HRQoL) during that period [30]. The values of the weights typically lie on a scale between zero (equivalent to death) and one (equivalent to full health), although negative values for states rated worse than dead are possible. This captures effects on both the quality and quantity of life used in assessment of health interventions in the UK health service [31]. The use of the QALY enables comparisons of the relative cost-effectiveness of interventions to be made across a range of conditions.

*Warwick-Edinburgh Mental Well-Being*. It was assessed using the short version of the Warwick-Edinburgh Mental Well-Being scale which consists of 7 items to assess mental wellness [32].

*Illness Perception*. This was assessed using the Brief Illness Perception Score to assess the cognitive and emotional perceived illness [33].

*Health Status Measure*. It was assessed using the physical and mental health components of the SF12, which is a validated measure of overall health and daily activities [34].

*Resource Use*. It is the self-reported service use of family doctor and hospital care.

*Haemoglobin A1c Values*. Haemoglobin A1c values were extracted from the medical records. The closest available readings prior to date of randomisation and after 7 months were taken.

### 2.4. Sample Size

The primary aim of this pilot trial was to inform the design of a subsequent large-scale randomised controlled trial. Accordingly, the sample size was set to provide sufficient data to make reasonably accurate estimates of the underlying recruitment rate, statistical properties of the outcome measures, and some indication that the intervention has benefit for patients. On this basis, the target sample was set at 120 participants, allowing percentage recruitment to be estimated with an error of at most plus/minus 9% and the standard deviations of outcome measures to be between 0.89 and 1.14 times the actual value, with 95% confidence. With regard to patient benefit, although not powered to provide convincing evidence for a treatment effect (i.e., *p* < 0.05 is unlikely to be found), low *p*-values on some of the major outcomes can be viewed as supporting the intervention's likely effectiveness. Allowing for 25% attrition, a follow-up sample of 90 patients would give 80% power to yield a *p* value (two-tailed test) of 0.15 or less given a moderate to large effect size of the intervention (Cohen's *d* of 0.5 or greater). The above calculations do not take account of clustering of outcome scores within practices but do give a general indication of sample adequacy.

### 2.5. Randomisation

Participants were randomly allocated on a 1 : 1 basis to either the LHT intervention or usual care. For each family doctor practice, a computer generated block randomisation list using block sizes of 2, 4, and 6 was produced by the statistician who was blind to treatment allocation. The sequence of treatment allocation was then sealed in opaque envelopes to be given to the participant from the research nurse once baseline data had been collected. Participants allocated to the intervention group were then given an appointment with the health trainer for commencement of the intervention. To avoid the risk of “contamination” between members of the same household if more than one person had diabetes, only 1 person was recruited per household. To maintain allocation concealment, the follow-up outcome data was collected by a different researcher at Keele University who was blind to treatment allocation.

### 2.6. Statistical Analysis

**Figure 1:**
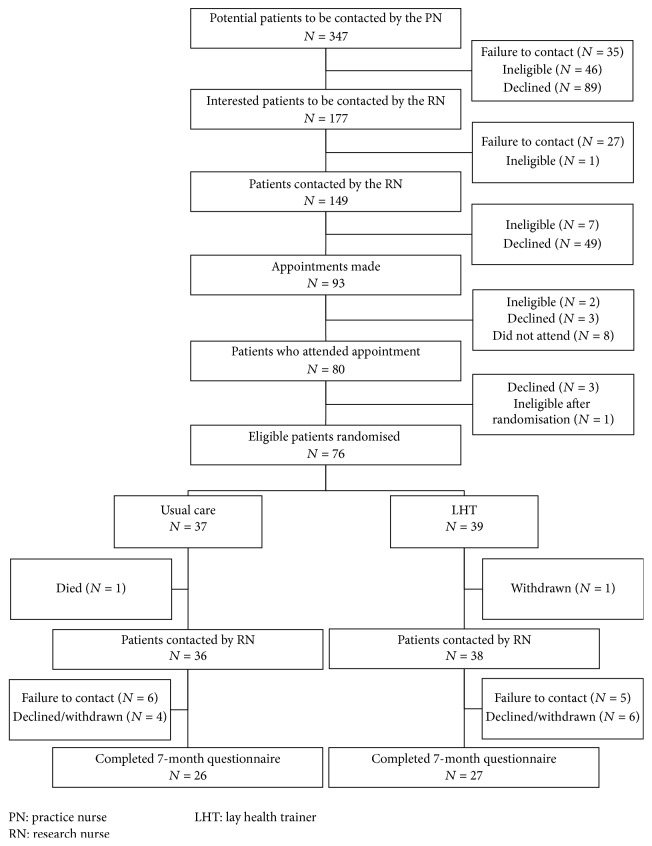
Consort diagram.

A consort flow diagram is presented (Figure 1). Descriptive statistics were used to assess whether the study had successfully recruited participants with low health literacy from a socioeconomic disadvantaged population. Descriptive statistics were used to assess balance of baseline characteristics between the trial arms and the distributional properties of each outcome measure. Analyses of the effectiveness of the LHT intervention at 7-month follow-up were conducted using an intention to treat approach within a linear regression framework. The primary analysis adjusted for baseline outcome scores only. To account for possible baseline differences on key prognostic factors, a sensitivity analysis was then performed adjusting also for age, gender, health literacy, family doctor practice, and length of time with diabetes. The assumptions of linear regression were verified. As this was an exploratory analysis looking solely for indications of effectiveness, imputation of missing values was not applied and all analyses were based on complete cases. All analyses were conducted in STATA v14.

### 2.7. Ethical Approval

Ethical approval was granted by the East Midlands, Derby National Research Ethics Service Committee on 16 August 2011, reference 11/EM/0294.

## 3. Results

### 3.1. Feasibility of Recruitment

Target recruitment was set at 120 patients; however, only 76 patients were recruited. Failure to reach target was due to the initial way potential participants were identified by the practice nurse with one family doctor practice failing to identify any potential participants.

Figure 1 is the consort flow diagram illustrating the flow of patients recruited to the trial. In summary, of the 290 eligible patients, 76 (26.2%) were randomised, 37 to usual care and 39 to the LHT arms. One ineligible Type 1 diabetic patient was randomised and was removed from analysis. The overall follow-up rate at 7 months was 69.7% (53/76); the follow-up rates in the usual care and LHT arms were 70.3% (26/37) and 69.2% (27/39), respectively, indicating drop-out was not related to trial allocation.

### 3.2. Descriptive Statistics

Patient characteristics are summarised in Table 1. There were some differences between the two groups; the LHT arm was slightly older, had more females, had less retirees, and had diabetes for a longer period of time. The majority of the patients had poor health literacy and had come from a socioeconomic disadvantaged population. 46 (60.5%) had less than adequate health literacy skill. Nationally, 20% of the UK population reside in each deprivation quintile. Compared to the rest of England, 75% of the study sample resided in the most deprived areas of England, higher than the expected national level of 40%.

### 3.3. Evaluation of Health Outcome Measures

Table 2 describes the distribution of each outcome at baseline and 7 months. With the exception of the EQ5D and Diabetes UK Score, the rest of the outcomes demonstrate good performance in having wide variation but only small floor or ceiling effects. The EQ5D and Diabetes UK Score show substantial floor and/or ceiling effects at baseline and/or 7 months: over 60% of patients have an EQ5D score in the top 20% of possible scores; and up to 40% have a Diabetes UK Score in the top 20% of possible scores. This suggests that the EQ5D and Diabetes UK Score may not be suitable outcomes to use in a further full trial as they have limited ability to detect change at the top end of the scale.

There were good indications that the LHT can improve patients' mental health; at 7 months, participants in the LHT (intervention) arm on average had a higher mental component score (mean difference between arms = 5.46, 95% CI: 0.02, 10.89, *p* = 0.049) and a less negative view on illness perception (−5.74, 95% CI: −11.19, −0.29, *p* = 0.040). Both of these results reached the conventional level of statistical significance of *p* < 0.05. In terms of direction of change, participants in the LHT (intervention) arm had improved patient self-care management, received more health services and checks, and had better QALY profile but had worse haemoglobin A1c (all nonsignificant, but with *p* values close to 0.2). However, there was less evidence for any impact on the physical health, well-being, and diabetes quality of life (Table 3). The pattern of results was essentially unchanged under sensitivity analysis adjusting for prognostic factors (Table 3), with the exception of haemoglobin A1c where the adjusted *p* value suggested little effect on HbA1c over the length of this study.

### 3.4. Resource Use

The intervention was associated with lower resource use across all categories at 7-month follow-up. Results are presented in Table 4. The inpatient stay was lower in the LHT arm, though there were very few responses. It was assumed in the first case that missing values were in fact zero. However, conducting an analysis on those who completed the questionnaire and provided values for the first and/or second stay showed consistent results (inpatient stay mean for first stay was 13 days in usual care and 5.67 in LHT intervention and 8.5 and 0 for second visit). All of these results were not significant at conventional levels.

## 4. Discussion

This was a pilot randomised controlled trial of a lay health trainer (LHT) intervention to encourage patients to make healthy lifestyle choices in their self-management of Type 2 diabetes. The trial focused on the feasibility of recruitment of patients with low health literacy and poorly controlled diabetes from a socioeconomically disadvantaged population and evaluating its effect on diabetes self-management with a preliminary assessment of cost-effectiveness of the LHT intervention.

### 4.1. Recruiting from Disadvantaged Populations

As expected, recruiting participants with low health literacy from a socioeconomically disadvantaged population was challenging and required alterations to our recruitment strategy. At the outset, we had made the decision not to use postal written information as we were particularly interested in recruiting patients with low health literacy for whom written information might be less accessible. We had felt that personal contact from the practice nurse when the patient attended for their regular review would be more appropriate. However, this method proved to be slower in recruiting patients than was expected. When the study team explored this with our recruiting practices, it appeared that there were two reasons in particular for this; first, amongst all the clinical tasks that she was performing, often the practice nurse would fail to remember to mention the study and, second, the patients who were in this most at risk group were poor attenders of their review appointments. When we changed our recruitment strategy to support the practice staff to directly contact eligible patients by telephone and discuss their potential participation in the trial, we were more successful in recruiting this disadvantaged population. This finding adds weight to the argument that it is not the particular population that is problematic but the failure to adopt recruitment strategies sensitive to contributing factors that may have an impact on participation [35]. With this method, as can be seen from the consort diagram in Figure 1, only 89 out 347 (25%) declined to be contacted further. However, the numbers of patients declining to participate once they spoke to the study team and failure to make contact with participants for follow-up were quite significant in this population.

Despite these substantial challenges, this method of recruitment was successful in recruiting a study population, 75% of whom were from the most deprived areas of England and over 60% had low health literacy (as measured by the NVS) [25]. This compares well to other studies of LHTs which were less successful in this aspect and tended to recruit more affluent populations [36].

### 4.2. Effect of Intervention

Given that this was a feasibility pilot trial and powered accordingly, nonetheless, these provisional results show that the LHT had a significant impact on the mental health of participants in the intervention arm, both in terms of the mental health component of the SF12 and in patients having a less negative self-perception of their condition. There may be a variety of reasons underlying this; research evidence suggests that patients with low health literacy can be especially anxious about medication use and dissatisfied with information that they receive about diabetes [37]. Additionally, other research has suggested that enhanced social support (signposted to or directly provided by the LHT) may improve diabetes self-care [38]. Furthermore, although not achieving conventional statistical significance in this small sample size, the results suggest that participants in the LHT arm had improved patient self-care management and received more health services and checks, all of which are likely to positively impact participants' mental health and their perceptions of their condition. This is supported by a relatively large increase in quality adjusted life years (QALYs) over a short seven-month period.

The LHT intervention in this pilot trial did not lead to improvements in physical health or blood-glucose control as measured by the HbA1c, but the sample was not powered to investigate changes in HbA1c and it is likely that an intervention of this nature, designed to improve patient self-management by encouraging patient behaviour change, would need longer than the short follow-up of this study to demonstrate an impact on physical health.

### 4.3. Preliminary Cost-Effectiveness

The LHT intervention was associated with lower resource use across all categories (primary and secondary care) at follow-up. While none of these differences were clinically significant, these results add weight to the possibility that the relatively minimal costs of the intervention may be offset by reductions in downstream costs. In addition, the intervention was associated with a better QALY profile than the control group. While this difference was small (and nonsignificant), it supports the general results of this study that the intervention may provide good value for money and may even save money while improving outcomes.

Research evidence on the cost-effectiveness of lay health advisors, which would include LHTs, is mixed, but an evidence synthesis by Carr et al. suggests that they can be cost-effective in chronic care and smoking cessation, both important for diabetes self-management [39].

### 4.4. Limitations of This Study

Despite relatively successful efforts to recruit a disadvantaged population of patients with poorly controlled diabetes and low health literacy, from socioeconomically deprived areas, there remains the possibility that the trial participants are still underrepresentative of those who are most disadvantaged and most at risk. Such individuals may be less motivated to respond to the LHT intervention and less willing to respond to supported self-management to improve their poorly controlled diabetes.

Being a pilot, the trial was not fully powered for the detection of intervention effects and the inclusion of a wide range of outcomes implies a high chance of one or more falsely significance results; hence, the findings on effectiveness must be treated as purely provisional until validated by further, larger, studies. A further limitation is the short length of follow-up. This is particularly relevant to interventions which are intended to lead to change in outcome measures through behaviour change, which will likely need a reasonable length of time to make an impact. This would need to be evaluated in a full-size RCT with longer follow-up.

### 4.5. What This Study Adds

This study adds to the body of evidence regarding recruiting disadvantaged participants, specifically those with low health literacy, living in socioeconomically disadvantaged areas. We would support recruitment strategies that keep written information to a minimum and recruit using personal contact by someone known to the potential participant. As previously mentioned, a future full-sized RCT would need to aim for longer follow-up of 12–18 months to be confident about sustained improvements in mental health and the possibility of improvements in patient self-management leading to significant improvements in physical health. As discussed in the results, follow-up in this study at 7 months was just under 70%, so collecting longer term follow-up data will be challenging and may require the use of other more innovative practices such as the use of text messaging and social media to collect data, keeping in mind health literacy limitations.

## 5. Conclusion

Despite the initial low response to recruitment using practice nurses, changes in our recruitment strategy led to this pilot trial recruiting the population that it set out to achieve. To our knowledge, this is the first pilot trial to provide evidence for recruiting patients with low health literacy from disadvantaged backgrounds and to demonstrate the feasibility of a LHT RCT in a primary care setting for this population that is usually excluded from RCTs by nature of their poor response to invites to research. Adding to this the likely cost-effectiveness of this relatively low-cost intervention to a population currently suffering a disproportionate burden of diabetes and diabetes-related complications, we would support a large, robust RCT to demonstrate the treatment effect and its sustained impact on health and health inequalities.

## Figures and Tables

**Table 1:** Baseline patient characteristics between usual care and health trainer.

Patient characteristic	Usual care *N* = 37	Health trainer *N* = 39
Age (mean (SD))	61.5 (10.1)	64.7 (11.2)
Sex		
Male	22 (59.5)	16 (41.0)
Female	15 (40.5)	23 (59.0)
Deprivation		
Most deprived	11 (29.7)	13 (33.3)
2nd most deprived	16 (43.2)	17 (43.6)
Mid-deprived	8 (21.6)	5 (12.8)
2nd least deprived	2 (5.4)	4 (10.3)
Least deprived	0 (0)	0 (0)
Employment status		
Paid work	13 (35.1)	15 (38.5)
Retired	17 (46.0)	15 (38.5)
Long-term sick/disabled	6 (16.2)	6 (15.4)
Seeking employment/volunteer work/looking after home or family	1 (2.7)	3 (7.7)
Marital status		
Never married	7 (18.9)	4 (10.3)
Married/civil partnership	21 (56.8)	21 (53.9)
Separated/divorced/widowed	9 (24.3)	14 (35.9)
Lives alone		
Yes	9 (24.3)	10 (27.0)
No	28 (75.7)	27 (74.0)
How long patient had diabetes (years)		
<5 years	11 (29.7)	4 (10.3)
≥5 years	26 (70.3)	35 (89.7)
Number of comorbidities		
0-1	8 (21.6)	12 (30.8)
2-3	18 (48.7)	21 (53.9)
4-5	11 (29.7)	6 (15.4)
Highest qualification obtained		
School level including O-level/CSEs/GCSEs/School certificate or none	9 (26.5)	21 (53.9)
A-level or vocational including NVQ/HNC/HND/professional qualification/other	22 (64.7)	16 (41.0)
University (first or higher education)	3 (8.8)	2 (5.1)
Health literacy		
Adequate	17 (46.0)	13 (33.3)
Inadequate	20 (54.0)	26 (66.7)
Socioeconomic status		
Higher managerial administration and professional occupations	9 (24.3)	10 (25.6)
Intermediate occupations	10 (27.0)	12 (30.8)
Routine and manual occupations	18 (48.7)	17 (43.6)
Feeling down, depressed, or hopeless (QOF depression screen 1)		
Yes	16 (43.2)	15 (38.5)
No	21 (56.8)	24 (61.5)
Little interest or pleasure in doing things (QOF depression screen 2)		
Yes	17 (46.0)	16 (41.0)
No	20 (54.1)	23 (59.0)

**Table 2:** Adequacy of outcome measures.

Outcome measure	Number of patients answering all scale items *N* (%)	Range of possible scores	Mean score (SD)	Range of observed scores	Number of patients with minimum possible score *N* (%)	Number of patients with maximum possible score *N* (%)	Number of patients scoring in the bottom 20% of possible scores *N* (%)	Number of patients scoring in the top 20% of possible scores *N* (%)
Baseline: SDSCAM	76 (100)	0, 7	3.83 (1.48)	0.22, 6.89	0 (0)	0 (0)	4 (5.3)	7 (9.2)
7 months: SDSCAM	52 (98.1)	0, 7	4.01 (1.24)	0.89, 6.78	0 (0)	0 (0)	1 (1.9)	5 (9.6)
Baseline: SWEMWBS	76 (100)	7, 35	22.93 (5.30)	13.30, 35.00	0 (0)	3 (4.0)	0 (0)	8 (10.5)
7 months: SWEMWBS	52 (98.1)	7, 35	22.81 (4.22)	7.00, 30.70	1 (1.9)	0 (0)	1 (1.9)	2 (3.8)
Baseline: PCS	76 (100)	0, 100	36.90 (10.64)	9.94, 56.15	0 (0)	0(0)	6 (7.9)	0 (0)
7 months: PCS	53 (100)	0, 100	35.36 (13.04)	7.89, 56.15	0 (0)	0 (0)	5 (9.4)	0 (0)
Baseline: MCS	76 (100)	0, 100	45.44 (12.76)	15.36, 65.63	0 (0)	0 (0)	3 (9.2)	0 (0)
7 months: MCS	53 (100)	0, 100	49.16 (12.12)	17.36, 74.12	0 (0)	0 (0)	1 (1.9)	0 (0)
Baseline: DQL	76 (100)	0, 100	34.12 (24.05)	0, 100	2 (2.6)	2 (2.6)	19 (25)	5 (6.6)
7 months: DQL	48 (90.6)	0, 100	39.06 (25.98)	0, 100	2 (4.2)	1 (2.1)	9 (18.8)	4 (14.6)
Baseline: BIPS	76 (100)	0, 80	38.46 (12.80)	11, 63	0 (0)	0 (0)	3 (3.9)	0 (0)
7 months: BIPS	52 (98.1)	0, 80	38.33 (12.01)	12, 68	0 (0)	0 (0)	2 (3.8)	2 (3.8)
Baseline: DUKS	76 (100)	0, 9	6.97 (1.15)	3, 9	0 (0)	1 (1.3)	0 (0)	27 (35.5)
7 months: DUKS	52 (98.1)	0, 9	7.15 (1.24)	4, 9	0 (0)	8 (15.5)	0 (0)	21 (40.4)
Baseline: Hb1Ac	76 (100)	—	78.04 (15.17)	56, 121	—	—	—	—
7 months: Hb1Ac	61 (80.3)	—	72.64 (16.71)	41, 117	—	—	—	—
Baseline: EQ5D	76 (100)	−0.59, 1	0.59 (0.35)	−0.24, 1.00	0 (0)	12 (15.8)	0 (0)	47 (61.8)
7 months: EQ5D	52 (98.1)	−0.59, 1	0.64 (0.28)	−0.02, 1.00	0 (0)	7 (13.5)	0 (0)	31 (59.6)

Summary Diabetes Self-Care Measure (SDSCAM); Short Warwick-Edinburgh Mental Well-Being Score (SWEMWBS); SF-12 Physical and Mental Component Scores (PCS & MCS); Diabetes Quality of Life (DQL); Brief Illness Perception Score (BIPS); Diabetes UK Score (DUKS); EuroQuol Health questionnaire (EQ-5D).

**Table 3:** Effectiveness of the health trainer arm.

Outcome measure	Usual care		Lay health trainer		Mean difference adjusted for baseline 95% CI	*p* value	Sensitivity analysis: mean difference adjusted for baseline, gender, health literacy, PCP, and time with diabetes 95% CI	*p* value
	*N* = 26		*N* = 27					
	Baseline	7 months	Baseline	7 months				
SDSCAM: higher scores reflect better management of diabetes	4.00 (1.44)	3.85 (1.02)	4.00 (1.29)	4.18 (1.42)	0.33 (−0.21, 0.87)	0.224	0.33 (−0.24, 0.91)	0.249
PCS: higher scores reflect better physical function	35.90 (9.35)	35.68 (12.81)	35.42 (11.83)	35.06 (13.49)	−0.27 (−6.13, 5.60)	0.928	0.83 (−5.60, 7.26)	0.796
MCS: higher scores reflect better mental function	48.59 (9.73)	47.23 (11.08)	45.86 (13.57)	51.02 (12.98)	5.46 (0.02, 10.89)	0.049	6.61 (0.54, 12.68)	0.034
SWEMWBS: higher scores reflect better mental well-being	22.14 (4.39)	22.44 (2.86)	23.88 (5.59)	23.19 (5.27)	−0.17 (−2.13, 1.80)	0.865	−0.26 (−2.43, 1.92)	0.814
BIPS: higher scores reflect patients viewing their diabetes as more life threatening	33.46 (11.26)	38.76 (10.87)	39.26 (12.40)	37.93 (13.17)	−5.74 (−11.19, −0.29)	0.040	−5.91 (−11.89, 0.08)	0.053
DQL: higher scores reflect worse quality of life	28.21 (21.21)	39.02 (23.24)	33.70 (23.56)	39.10 (28.55)	−4.24 (−16.34, 7.87)	0.485	−3.13 (−16.58, 10.33)	0.641
DUKS: higher scores reflect patients were offered more health services to help manage their diabetes	7.27 (0.87)	6.96 (1.43)	7.19 (0.74)	7.33 (1.04)	0.40 (−0.27, 1.06)	0.233	0.29 (−0.41, 0.98)	0.414
HbA1c: higher scores reflect worse control of blood sugars	78.38 (13.86)	66.19 (18.60)	77.37 (16.54)	72.88 (14.82)	5.17 (−2.53, 12.88)	0.183	1.42 (−7.18, 10.02)	0.740
EQ5D: higher scores reflect better health	0.67 (0.26)	0.61 (0.27)	0.56 (0.36)	0.68 (0.28)	0.10 (−0.03, 0.24)	0.135	0.13 (−0.02, 0.27)	0.082

Summary Diabetes Self-Care Measure (SDSCAM); Short Warwick-Edinburgh Mental Well-Being Score (SWEMWBS); SF-12 Physical and Mental Component Scores (PCS & MCS); Diabetes Quality of Life (BDQL); Brief Illness Perception Score (BIPS); Diabetes UK Score (DUKS); EuroQuol Health questionnaire (EQ-5D).

**Table 4:** Resource use by group (number of contacts) at 7 months based on complete cases.

	Usual care *N* = 26 Mean (SD)	Lay health trainer *N* = 27 Mean (SD)	Unadjusted difference (95% CI)	*p* value
Inpatient (number of nights)^*∗*^	3.12 (8.34)	1.26 (3.40)	−1.86 (−5.35, 1.63)	0.291
A&E attendance	0.54 (1.03)	0.46 (0.86)	−0.08 (−0.60, 0.45)	0.771
Outpatient visits	1.19 (1.86)	0.65 (0.89)	−0.54 (−1.35, 0.27)	0.188
GP at surgery	2.58 (2.16)	1.65 (1.60)	−0.92 (−1.98, 0.13)	0.086
GP at home	0.04 (0.20)	0 (0)	−0.04 (−0.12, 0.04)	0.322
Practice nurse	2.12 (2.10)	1.73 (1.15)	−0.38 (−1.33, 0.56)	0.417

^*∗*^Based on the assumption that missing values were zeros.
